# Soil Acidobacterial community composition changes sensitively with wetland degradation in northeastern of China

**DOI:** 10.3389/fmicb.2022.1052161

**Published:** 2022-12-23

**Authors:** Xin Sui, Beat Frey, Libin Yang, Yingnan Liu, Rongtao Zhang, Hongwei Ni, Mai-He Li

**Affiliations:** ^1^Engineering Research Center of Agricultural Microbiology Technology, Ministry of Education, Heilongjiang University, Harbin, China; ^2^Heilongjiang Provincial Key Laboratory of Ecological Restoration and Resource Utilization for Cold Region, School of Life Sciences, Heilongjiang University, Harbin, China; ^3^Snow and Landscape Research WSLSwiss Federal Institute for Forest, , Birmensdorf, Switzerland; ^4^Institute of Nature and Ecology, Heilongjiang Academy of Sciences, Harbin, China; ^5^Heilongjiang Academy of Forestry, Harbin, China; ^6^Key Laboratory of Geographical Processes and Ecological Security in Changbai Mountains, Ministry of Education, School of Geographical Sciences, Northeast Normal University, Changchun, China; ^7^School of Life Science, Hebei University, Baoding, China

**Keywords:** Sanjiang plain, soil bacterial diversity, β diversity, high-throughput sequencing, forest, community structure

## Abstract

Acidobacteria are a major component of the soil bacteria and are conducted for many soil functions, and the soil Acidobacterial structure and diversity are affected by climate changes and human activities. However, soil Acidobacterial structure and diversity in wetland ecosystems are still limited recognized. The current study aimed to study the Acidobacterial community and diversity in relation to soil environmental factors along a typical degradation series from primitive wetland to forest in a representative fresh wetland in northeastern China. In this research, we assessed the soil Acidobacterial community composition, using Illumina MiSeq sequencing along a typical degradation series from primitive wetland to forest in a representative fresh wetland in northeastern China. The soil physico chemical properties changed significantly among the eight degrade stages (*p* < 0.05). The α diversity index (Shannon and Chao1 index) of soil Acidobacteria changed significantly between different degradation stages (*p* < 0.05). Principal Coordinates Analysis (PCoA) revealed that the soil acidobacteiral communities obviously separated into wetland group and forest group. The most abundant subgroups of Acidobacteria accounted for 31% (Gp1), 5% (Gp2), 12% (Gp3), 2% (Gp4), 5% (Gp6), and 2% (Gp7) in soils within eight successional series. The compositions of soil Acidobacteria in wetland stages were significantly affected by soil moisture content, soil total nitrogen and available nitrogen contents, while those in forest stages were significantly driven by soil pH, soil organic carbon, total nitrogen, available phosphorus and soil moisture content. Our results indicated that the soil Acidobacterial community was mainly structured by soil physico chemical parameters, and wetland degradation towards forests will greatly influence the soil Acidobacterial structure and thus the wetland functions.

## Introduction

Acidobacteria are a major bacterial phylum recently classified based on research of molecular ecology and widely distributes in the natural soil and the proportion of Acidobacteria is almost same with Proteobacteria (Janssen, 2006; Wang et al., 2010, 2016). Acidobacteria plays a crucial role in soil element cycle and ecological function (Ward et al., 2009; Liu et al., 2014), such as cellulose, methylcellulose, xylan and pectin (Pankratov et al., 2008; Eichorst et al., 2011). Blöthe et al. (2008) found that Acidobacteria can participate in iron circulating. Radajewski et al. (2002) also indicated Acidobacteria may be participated in the carbon metabolism. However, due to its difficult cultivation, the ecological functions of Acidobacteria are still very limited.

Acidobacteria can be divided into 8 different subgroups from Gp1 to Gp8, most of which are acidophiles (Wang et al., 2010). The composition of vegetation has an important influence on the composition of Acidobacteria. For example, Naether et al. (2012) used primers 31F/1492R to amplify and construct a clone library of Acidobacteria in soils collected from different grasslands and forests in Germany. They found that Acidobacteria in forest soils were mainly Gp1 (relative abundance: 26–85%), Gp6 (1–41%), Gp3 (7–11%), Gp4 (6%) and Gp5 (12–13%), while those in grasslands were mainly Gp1 (59–62%), Gp4 (8–20%), Gp5 (3–17%), and Gp17 (6–7%) (Naether et al., 2012). Fierer et al. (2011) used high-throughput sequencing and cloning methods to investigate the distribution of Acidobacteria in 87 forest soils collected from North and South America and found Acidobacteria accounted for 30.9% of the total bacteria identified, and there were 8,600 Acidobacterial genotypes, and the relative abundance of different Acidobacterial subgroups ranged from 2.4 to 78.5% following a decreasing order of Gp4 > Gp1 > Gp3 > Gp2 > Gp6. Zhang et al. (2014) used high-throughput sequencing technology and found that Acidobacteria in forest soils had at least 4,480 OTUs, of which Gp1, Gp2, Gp3, Gp4, and Gp6 were the dominant subgroups accounting for 85% of the total Acidobacteria. Wang et al. (2010) used high-throughput sequencing technology and discovered Gp1, Gp2, Gp3, and Gp5 were the principal Acidobacteria in forest soils. Liu et al. (2014) and Yao et al. (2021) found that Gp1, Gp3, Gp4 and Gp6 were the vital Acidobacteria in mollisol of northeastern China. For wetland ecosystems, Pankratov et al. (2008) studied the Acidobacteria of Sphagnum-dominated wetlands of West Siberia and European North in Russia and found that Gp1 and Gp3 are the dominant Acidobacteria in wetland soils. Sui et al. (2019) found that Gp1, Gp2, Gp3, Gp6, and Gp7 were the dominant Acidobacteria in the wetland soil.

The composition of Acidobacteria correlates with soil physicochemical properties. Some previous studies reported soil pH was a vital environmental factor affecting the Acidobacterial composition. For example, Fierer et al. (2011) indicated that the composition of Acidobacteria was affected significantly by soil pH, and the same results were also reported in other studies (Männistö et al., 2007; Jones et al., 2009; Griffiths et al., 2011; Navarrete et al., 2013). However, Liu et al. (2014) found the composition of Acidobacteria did not correlate with soil pH. Fierer et al. (2011) also proved soil organic carbon and soil C/N ratio were the key soil environmental factors affecting the soil Acidobacterial composition. The occurrence of these results perhaps related to the variations in the response of different subgroups of Acidobacteria, or even different Acidobacterial species in same subgroup, to soil environmental factors. The distribution of phylum of Acidobacteria, different subgroups of Acidobacteria, and even different Acidobacterial genotypes (OTUs) in the same subgroup varies greatly in soil, and the distribution of Acidobacteria is regulated by a variety of environmental factors. Therefore, we speculate that the compositions of soil Acidobacteria are varied among different vegetations and soil properties according to the studying ecosystems, and more work is needed to discover the compositions and diversity of Acidobacterial communities in soils.

Through an Illumina MiSeq Sequence technique, we examined the composition and diversity of bacterial and fungal communities across the wetland gradient types in Sanjiang plain (Sui et al., 2021), which is one of the most important wetland ecosystem for northeast of China (Wang et al., 2022). Our work revealed that Acidobacteria was the most abundant bacterial phylum in the wetland soils of Sanjiang plain (Sui et al., 2019, 2021). Moreover, we also found that wetland degradation resulted in the composition of Acidobacteria in forest type was higher than that of wetland type and the driving physicochemical parameters were also significant different (Sui et al., 2021). Given the knowledge of soil Acidobacteria in most studies were retrieved from high-throughput sequencing data using universal bacterial primers (Lauber et al., 2008; Jones et al., 2009; Liu et al., 2014; Zhang et al., 2014), we worried that some of the valuable information about Acidobacteria in soils had not been well described.

Due to the serious agricultural activities and global climate changes, the water level of the wetlands in the Sanjiang Plain continues to decline, leading to significant degradation of the original wetlands. Sui et al. (2021) reported that wetland degradation resulted in the composition of Acidobacteria in forest type was higher than that of wetland type. Although the research on niche and lifestyle for the phylum Acidobacteria has been studied (Hartmann et al., 2017), but we still lack deep understand on the differential response at subgroup level to changes in soil physicochemical parameters, and how changes of Acidobacterial subgroups with vegetation change. In Sanjiang Plain Field Experiment Platform, there are original natural wetland (NW), shrub-invaded wetland (IW), shrub-dominant wetland (DW), wetland edge (EW), young-*Betula* forest (YB), mature-*Betula* forest (MB), *Populus* and *Betula* mixed forest (PB), and conifer forest (*CF*) within a small distance, and the soil physicochemical properties in different degradation stages also changed significantly. This provides a unique base for us to study the impact of wetland degradation on the composition and diversity of soil Acidobacteria. Therefore, we investigated the composition of Acidobacteria at eight stages of wetland degradation in particular of the transition from wetland types to forest types. We hypothesized that: (1) the subgroups of Acidobacteria in soils change with wetland degradation from wetland (wetter) to forests (dry); and (2) soil physicochemical properties differently affect Acidobacterial structure between wetland and forests due to changes in soil water content. This study will provide basic data for further explaining the ecological functions of Acidobacteria in soil and also for in-depth revealing of the function of Acidobacteria in the element cycle in the process of wetland degradation and wetland ecosystems.

## Materials and methods

### Experimental site and soil sampling

This study was performed on in the Honghe National Nature Reserve (47°35’N, 133°31′E) of Sanjiang plain, China (Supplementary Figure S1). The mean annual temperature and precipitation is approximately and 1.9°C and 560 mm, respectively. Eight vegetation types were selected along slopes from the real wetland to the degraded wetlands for this study: i.e. NW, IW, DW, EW, YB, MB, PB, and *CF* (Supplementary Table S1).

In 2016, soils were collected from the eight vegetation types. Each vegetation type set three plots (20 m × 20 m). Ten to fifteen soil samples along an S-shaped path (0–20 cm) were sampled, using a sterile soil drill (5 cm in diameter, 20 cm deep) after removing the litter layer. The soil samples in each plot were pooled and mixed into one soil. The soil samples were then immediately transferred in refrigerator to keep at 4°C. The soil samples were sieved (2 mm mesh) to remove stones, roots and other debris, and divided into two parts-one was stored at −60°C for sequencing, and the other one was air-dried to conduct physicochemical analyses. For the above ground plant’s diversity, the details and Shannon diversity were shown in the Supplementary Table S1 and the methods were in our previous study (Sui et al., 2021).

The soil physicochemical properties were described in our previous study (Sui et al., 2021). Briefly, the soil pH was measured using a pH meter and soil to water ratio of 1:2.5 w/v. Soil organic carbon and total nitrogen were measured using an elemental analyzer (Elementar, Langenselbold, Germany). Available nitrogen was examined with a continuous flow analysis (SAN++, Skalar Analytical, Netherlands). Soil moisture content (MC) measured gravimetrically. Total phosphorus was measured with a spectrophotometer. Available phosphorus was measured using a colorimetric method upon extraction with 0.5 M NaHCO_3_.

### Soil DNA extraction and PCR amplification

Soil total DNA was extracted using a MOBIO-12888 soil DNA Isolation Kit (Mo Bio Laboratories, Carlsbad, CA, United States). DNA quantity and quality were first detected by a garose gel electrophoresis (1%) and then detected by using a NanoDrop ND-1000 spectrophotometer (Thermo Fisher Scientific Waltham, MA, United States). The special primers ACIDO (5′GCTCAGAATSAACGCTGG3′)/342r(5′CTGCTGCSYCCCGTAG3′) (~336 bp) were selected to amplify the Acidobacterial region (Lee and Cho, 2011). The PCR amplification system was conducted in a 25 μl reaction systems, consisted of 12.5 μl of PCR mix (Invitrogen, Shanghai, China), 1.5 μl of forward and reverse primers (10 μmol·L^−1^), 1 μl of DNA template (100 ng·L^−1^), and enough ultrapure water (ddH_2_O) to reach a 25 μl reaction volume. The amplification program were the following: pre-denaturation at 95°C for 8 min, 30 cycles of denaturation at 95°C for 60 s, annealing at 55°C for 60 s, and extension at 72°C for 60 s, and finally an extension step at 72°C for 20 min. The PCR products were inspected by 2% agarose electrophoresis, and the PCR products were purified with the AxyPrep DNA purification kit (Axygen Biosciences, Union City, CA, United States). Three independent PCR replicates per sample and then three PCR samples were pooled at equal amounts and paired-end sequenced on the Illumina MiSeq v3 platform (2 × 300 bp).

### Bioinformatics

QIIME Pipeline (version 1.8.0) was used to conduct the raw fasta sequences. Forward and reverse sequences were merged using the PEAR software (version 0.9.8). The sequences were removed if the mean quality score < 20 or the length < 200 bp and the ambiguities sequence were also removed by using the Trimmomatic (V0.33) software^1^ (DeSantis et al., 2006). The chimeras were removed by Usearch (version7.1,^2^) (Edgar et al., 2011). Exact barcode matching was implemented, which allowed for a two-nucleotide mismatch during primer matching by using the QIIME Pipeline (version 1.8.0). The obtained operational taxonomic units (OTUs) were clustered by Uprase at similarity threshold of 97% (Li, 2015) and the taxonomy were annotated to the SILVA database (v138.1) (Quast et al., 2013). If the OTU did not belong to plylum Acidobacteria, the OTU will be removed before next analysis. Before further analysis of α diversity, the reads were normalized according to the lowest number of reads for a single soil sample.

### Statistical analyses

The α diversity index were performed on QIIME1 platform at OTU level. The difference of Soil physicochemical properties, PD index, OTU, Chao1 and Shannon index in eight vegetation types were calculated by one-way ANOVA and Duncan test at a 0.05 significance level by using the software SPSS 17.0 software (SPSS Inc., Chicago, IL, United States). Principal Coordinates Analysis (PCoA) was also performed by using OTU table in the package of “vegan” R software (version 3.3.0; R Development Core Team, 2006) based on the Bray–Curtis dissimilarity (Hartmann et al., 2017; Frey et al., 2021). Permutational Multivariate Analysis of Variance (PERMANOVA) was performed in the “microeco” package of R software (version 3.3.0; R Development Core Team, 2006). Heatmap was generated using the relative abundance of Acidobacterial subgroups in R according to Frey’s method (Frey et al., 2016). The correlation heatmap between the soil physicochemical properties and soil Acidobacterial subgroups were performed in the “microeco” package of R software (version 3.3.0; R Development Core Team, 2006). Mantel test was also used to analyze at the wetland group and forest group and all soil Acidobacteria with soil physicochemical properties by using the “vegan” package of R software (version 3.3.0; R Development Core Team, 2006).

## Results

### Soil physicochemical properties in eight vegetation types

All the soil physiochemical properties changed significantly (*p* < 0.05) between the eight vegetation types (Table 1). MC was higher for the wetland group than for the forest group (Table 1). Soil pH was between 5.47 (PB) and 5.75 (NW) (Table 1). SOC was between 27.82 (*CF*) and 57.54 g/kg (DW) (Table 1). The TN was between 2.58 (*CF*) and 7.62 g/kg (NW). AN was between 165.86 (PB) and 455.25 mg/kg (NW). TP was between 0.32 (YB) and 6.36 mg/kg (NW). AP was between 25.18 (DW) and 51.99 mg/kg (YB).

**Table 1:** Soil physicochemical properties and soil enzymes in the eight vegetation types along a successional gradient in a degraded wetland.

Type	Soil moisture	pH	Total organic carbon (g/kg)	Total nitrogen (g/kg)	Available nitrogen (mg/kg)	Total phosphorus (mg/kg)	Available phosphorus (mg/kg)
NW	**52.79 ± 2.00a**	*5.47 ± 0.04d*	56.43 ± 3.13a	**7.62 ± 0.12a**	**455.25 ± 17.06a**	**6.36 ± 1.17a**	26.34 ± 1.05c
EW	23.52 ± 1.63d	5.58 ± 0.08bcd	56.09 ± 2.17a	3.00 ± 0.10e	214.47 ± 28.711d	0.52 ± .05c	26.45 ± 2.15c
IW	44.70 ± 2.07b	5.52 ± 0.03 cd	38.82 ± 3.33b	5.56 ± 0.11b	381.53 ± 23.97c	0.60 ± .11c	26.09 ± 2.50c
DW	35.62 ± 1.46c	5.69 ± 0.02ab	**57.54 ± 2.37a**	4.75 ± 0.02c	418.34 ± 10.88 ac	5.18 ± .19a	*25.18 ± 1.97c*
YB	20.07 ± .80de	*5.47 ± 0.03d*	37.88 ± 1.65b	4.28 ± 0.32d	329.55 ± 11.09b	*0.32 ± .06c*	**51.99 ± 2.13a**
MB	21.92 ± 1.83de	5.66 ± 0.02abc	36.49 ± 1.07b	7.25 ± 0.07a	437.61 ± 12.02 ac	0.62 ± .01c	43.15 ± 1.38b
PB	*16.09 ± .54e*	**5.75 ± 0.01a**	57.10 ± 2.34a	4.96 ± 0.16c	*165.86 ± 13.47d*	0.48 ± .13c	49.23 ± 4.35b
*CF*	17.18 ± 1.26e	5.58 ± 0.02bcd	*27.82 ± 2.14c*	*2.58 ± 0.17e*	189.12 ± 5.07d	2.43 ± .04b	29.60 ± 1.00c

Statistical significance (One-way ANOVA, *p* < 0.05) is indicated by different superscript letters in the same column. In each column, the largest value is shown in bold and the smallest value is shown in italics. Vegetation types: original natural wetland (NW), wetland edge (EW), shrub-invaded wetland (IW), shrub-dominated wetland (DW), young-*Betula* forest (YB), mature-*Betula* forest (MB), *Populus* and *Betula* mixed forest (PB), and conifer forest (CF).

### Acidobacterial α- and β-diversities

The Shannon diversity index, Chao1 index and OTU and PD index of soil Acidobacteria differed significantly (one-way ANOVA, *p* < 0.05) among the eight vegetation types (Table 2). The Shannon diversity index was between 4.8 (IW) and 5.7 (EW), and the Chao1 index was between 823 (MB) and 1032 (EW) (Table 2), and the OTUs was between 732 (MB) and 981 (EW) (Table 2). The PD index was between 112.4 (*CF*) and 153.1 (NW).

**Table 2:** Soil Acidobacterial α-diversity along a degraded wetland in Sanjiang plain.

Types	OTU	Shannon	Chao1	PD
NW	924.0 ± 10b	5.0 ± 0.01d	1032.6 ± 23a	153.1 ± 0.66a
IW	801.3 ± 13d	4.8 ± 0.01f	905.7 ± 28d	137.1 ± 1.98c
DW	822.0 ± 23d	5.0 ± 0.01 cd	959.6 ± 20bc	142.6 ± 0.39b
EW	981.7 ± 24a	5.7 ± 0.01a	1079.7 ± 40a	152.8 ± 2.14a
YB	742.3 ± 18e	5.1 ± 0.04bcd	867.6 ± 30de	117.2 ± 2.22d
MB	732.3 ± 15e	4.9 ± 0.08e	823.3 ± 21e	117.6 ± 2.06d
PB	877.0 ± 20c	5.1 ± 0.01b	977.5 ± 37b	120.7 ± 3.47d
*CF*	799.3 ± 8d	5.1 ± 0.04bc	918.3 ± 30 cd	112.4 ± 0.97e

Different lowercases indicate statistical significance identified by the Duncan test at *p* < 0.05.

**Figure 1:**
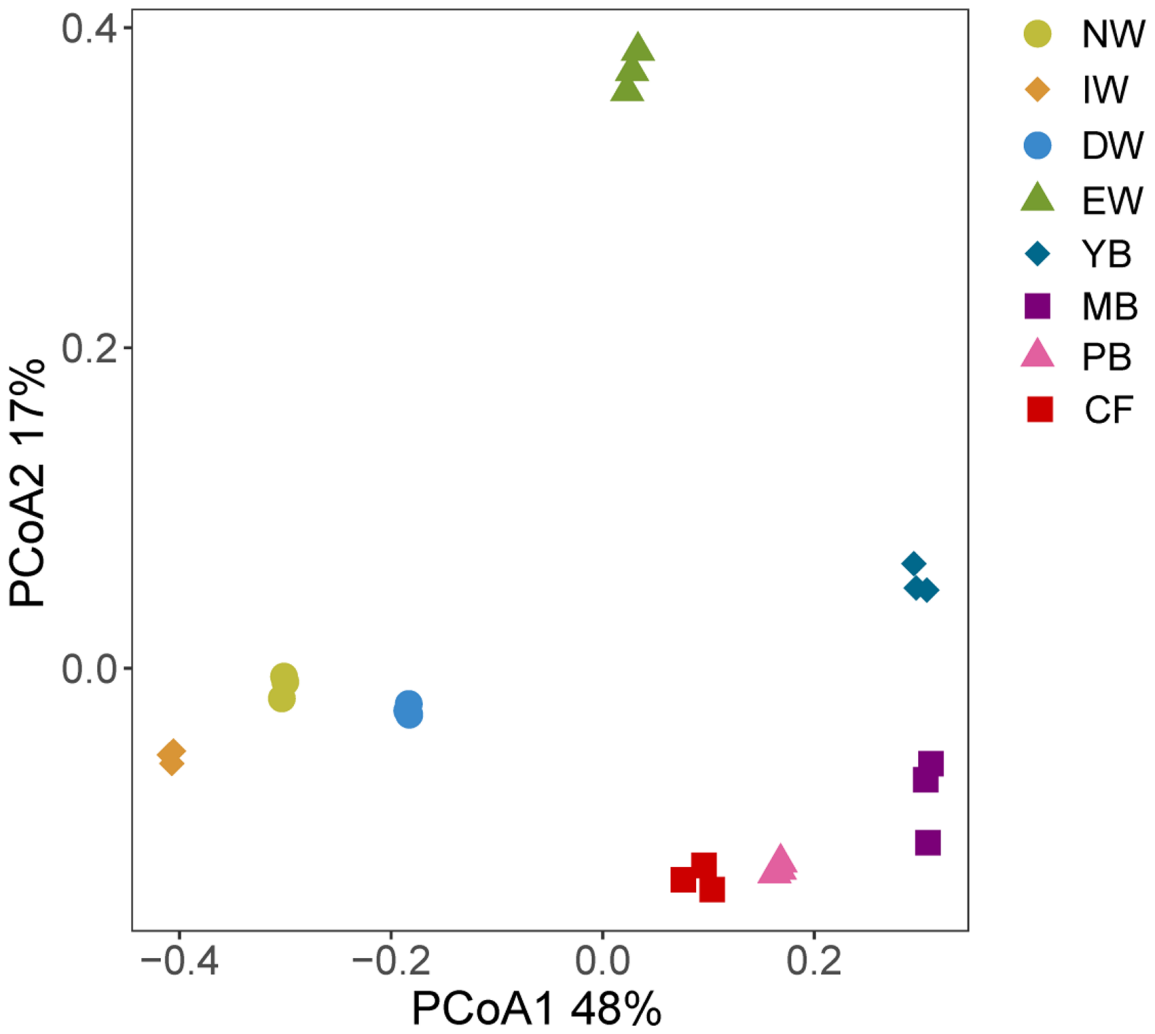
PCoA analysis of eight degradation vegetation types in Sanjiang plain. Vegetation types: original natural wetland (NW), shrub-invaded wetland (IW), w shrub-dominated wetland (DW), etland edge (EW), young-*Betula* forest (YB), mature-*Betula* forest (MB), *Populus* and *Betula* mixed forest (PB), and conifer forest (*CF*).

The PCoA separated the Acidobacterial community found in the eight vegetation types into three significantly different groups depending on their similarity, i.e., an wetland group (NW, IW, DW), a forest group (YB, MB, PB, and *CF*) and EW group (Figure 1; Table 3).

**Table 3:** Adonis analysis of the difference or similarity in Acidobacterial community between the wetland group and forest group in a degraded wetland of Sanjiang plain.

Vegetation	Sum of Sqs	Mean Sqs	*F*	*R* ^2^	*p*
All habitat	3.14	0.44	134.24	**0.98**	0.001
Wetland group versus forest group	1.25	1.25	14.16	**0.39**	0.001

The bold vales indicated that the significant difference at 0.05 level.

### Composition of soil Acidobacterial communities

**Figure 2:**
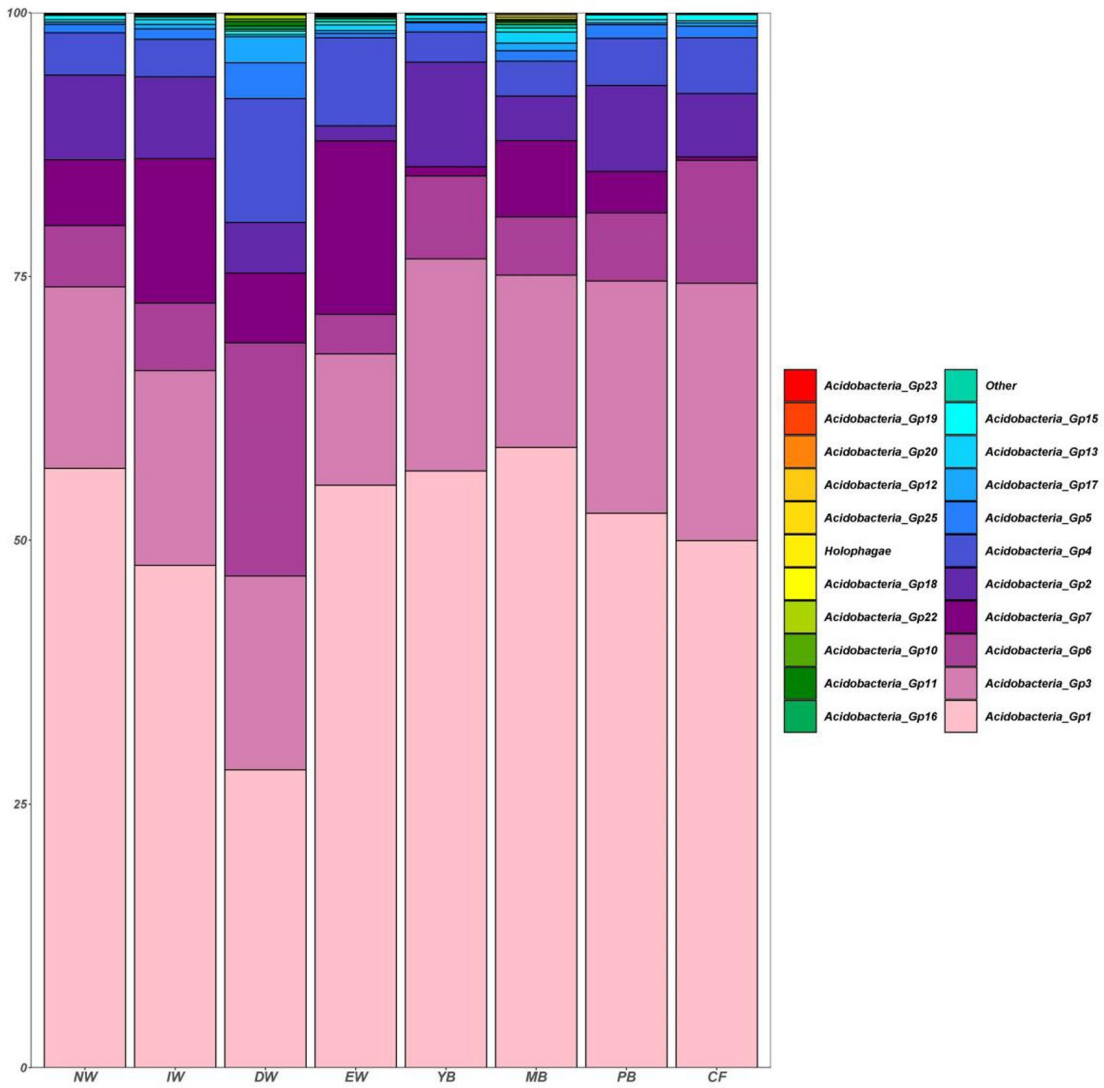
Histogram of the relative abundance of soil Acidobacterial communities in a degraded wetland.

**Figure 3:**
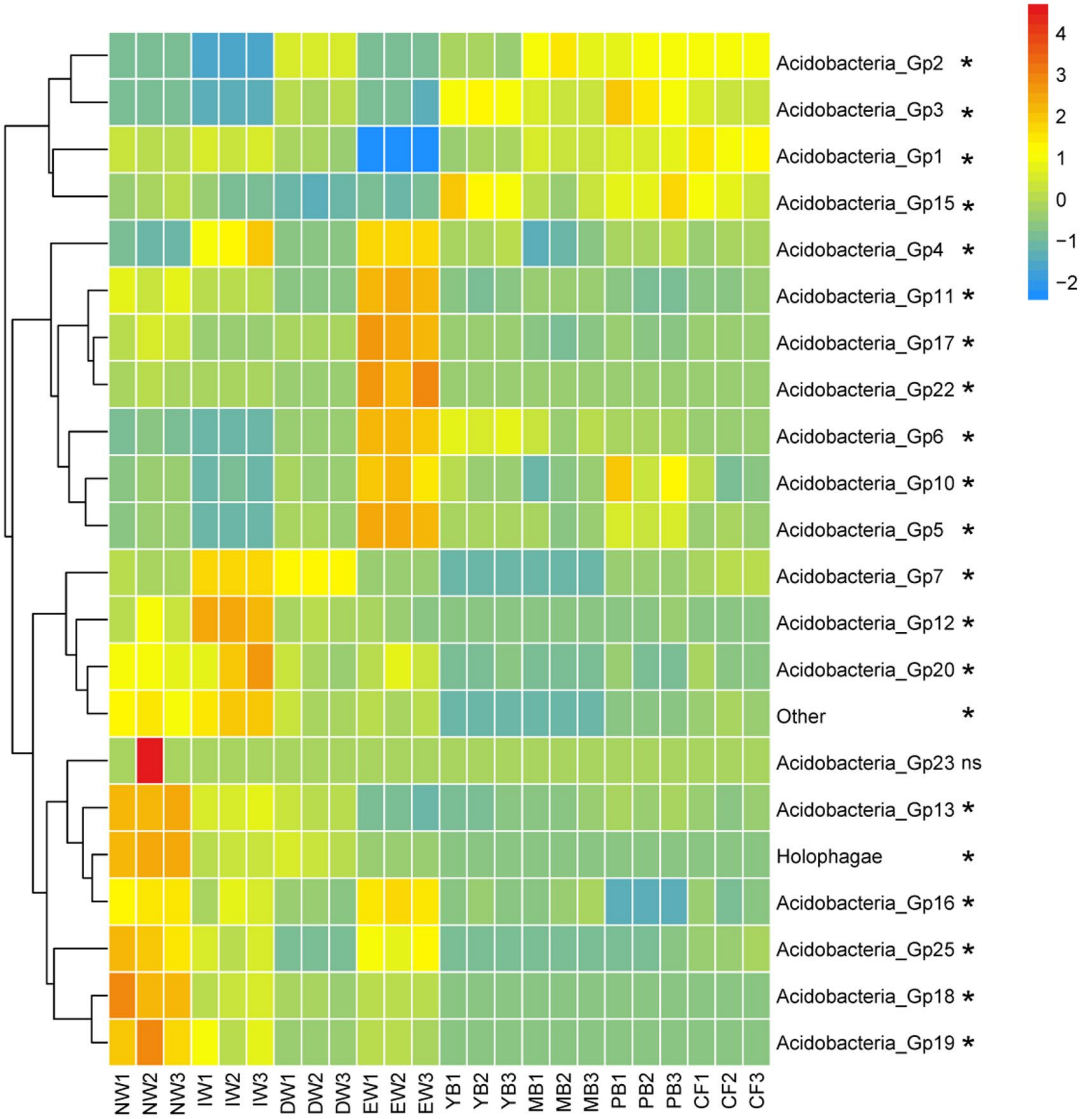
Heatmaps of Acidobacterial composition differences in different vegetation types in Sanjiang plain. *means one-way ANOVA significant at *p <* 0.05. The color gradient (red, yellow, blue) represents the relative abundance of the soil Acidobacteria from high to low in the different vegetation types.

A total of 1,545 Acidobacterial OTUs belonging to 22 subgroups were detected, following a decreasing order of relative abundance Gp1 > Gp3 > Gp7 > Gp6 > Gp2 > Gp4 > Gp13 > Gp5 >Gp17 > Gp15 > Other>Gp18 > Gp16 > Holophagae>Gp11 > Gp25 >Gp10 > Gp22 > Gp19 > Gp12 > Gp20 > Gp23 (Figure 2). Moreover, all the Acidobacterial subgroups, except Gp23, varied significantly among the eight vegetation types (Figure 3, *p* < 0.05).

The relative abundance of Gp7, Gp12, Gp20, Gp23, Gp13, Holophagae, Gp16, Gp25, Gp18, Gp19 in the wetland group (NW, IW, and DW) was higher than those in the forest group (YB, MB, PB and *CF*) (Figure 3). The relative abundance of Gp2, Gp3, Gp1, Gp15 in the wetland group (NW, IW and DW) was smaller than those in the forest group (YB, MB, PB and *CF*) (Figure 3). The relative abundance of Gp4, Gp11, Gp17, Gp22, Gp6, Gp10, Gp5 in EW was higher than those in the wetland group (NW, IW and DW) and forest group (YB, MB, PB, and *CF*) (Figure 3).

### The relationships between soil physicochemical properties and soil Acidobacterial communities

Mantel tests (Table 4) indicated that soil AP and MC were the key soil physicochemical properties affecting the soil Acidobacterial communities in all vegetation types (Table 4). But for wetland types, the soil TN, AN, and MC were the key soil physicochemical properties affecting the soil Acidobacterial communities, and for forest types, soil pH, SOC, AN, AP, MC, and TP significantly affected the soil Acidobacterial community (Table 4).

**Table 4:** Mantel test to determine the correlations between the environmental variables and acidobacterial community compositions at two habitats of wetland and forest.

Variables	Wetland group		Forest group		All samples	
	*r*	*p*	*r*	*p*	*r*	*p*
pH	0.086	0.217	**0.393**	0.001**	0.068	0.120
SOC	0.058	0.235	**0.372**	0.004**	0.080	0.104
TN	**0.605**	0.004**	0.102	0.173	0.067	0.143
AN	**0.667**	0.009**	**0.607**	0.006**	0.080	0.081
TP	0.111	0.117	**0.348**	0.009**	0128	0.051
AP	−0.057	0.632	**0.252**	0.048*	**0.385**	0.003**
MC	**0.733**	0.001**	**0.232**	0.04*	**0.666**	0.001**

**p < 0.01, *p < 0.05. The bold value indicated the significant difference. SOC, soil organic carbon; TN, total nitrogen; AN, available nitrogen; TP, total phosphorus; AP, available phosphorus; MC, moisture content; Wetland group included NW, IW, DW, and EW; Forest group included YB, MB, PB, and CF.

**Figure 4:**
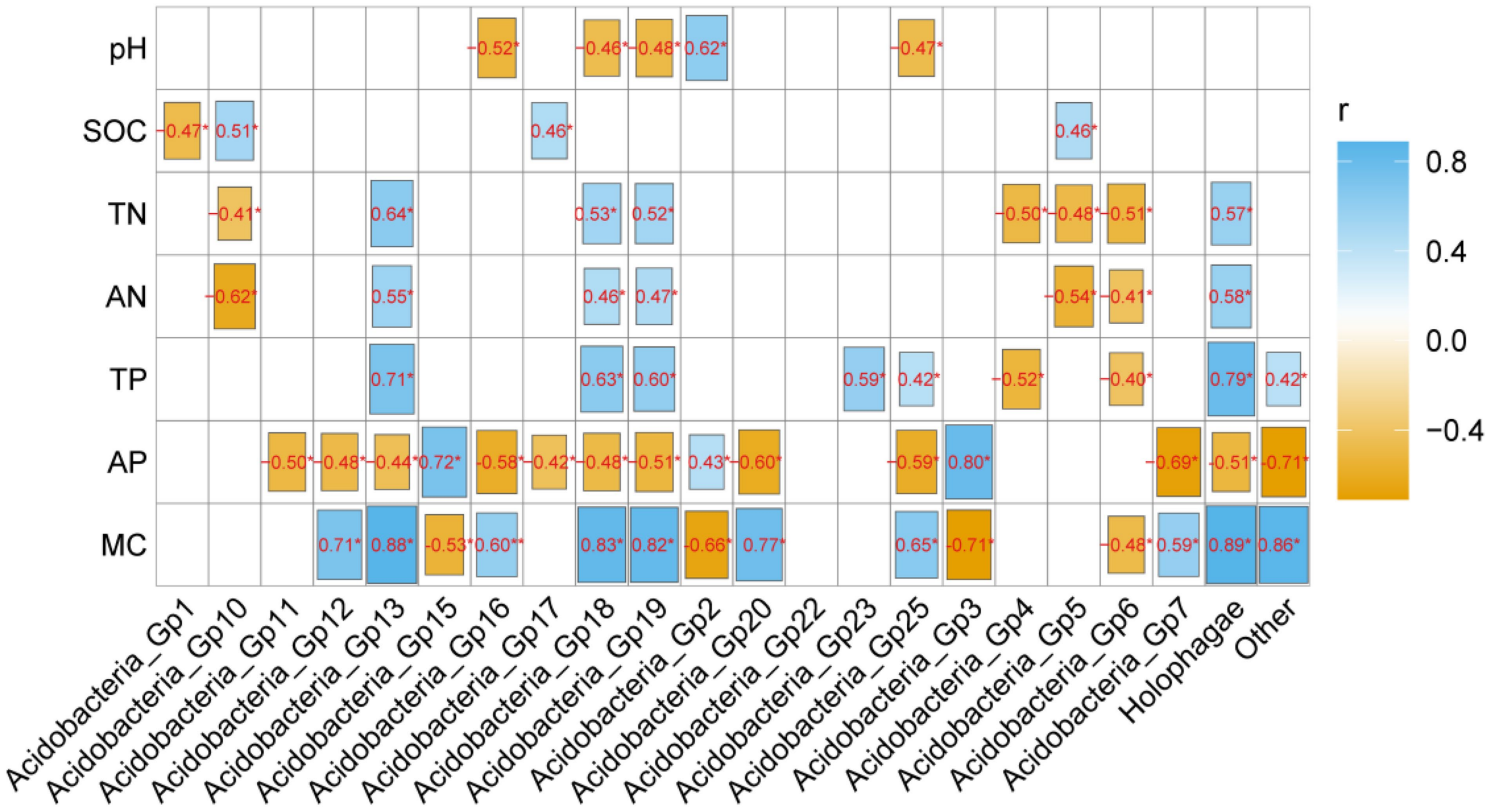
The correlation analysis of soil physicochemical properties and Acidobacterial subgroups. Blue colors indicate positive and yellow colors negative correlations. *, **, and*** mean 0.05, 0.01, and 0.001 levels, respectively.

The heamap analysis showed that Gp2 and Gp3 correlated significantly positively with soil AP (*p* < 0.05) and significantly negatively with MC (*p* < 0.05) and pH (*p* < 0.05), but the abundant Gp6 and Gp7 had the opposite correlation with these properties (Figure 4). Gp1 was significantly negative correlated with soil pH (*p* < 0.05). Moreover, Gp5 and Gp 17 were significantly positively correlated with SOC (*p* < 0.05), but the Gp5 significantly negatively correlated with TN (*p* < 0.05) and AN (*p* < 0.05), the Gp 17 significantly negatively correlated with AP (*p* < 0.05). Gp4 was significantly negatively positively with TP (*p* < 0.05) and TN (*p* < 0.05). Gp13 significantly positively correlated with MC (*p* < 0.05), AN (*p* < 0.05) and TN (*p* < 0.05), but negatively correlated with AP (*p* < 0.05) (Figure 4).

## Discussion

In line with our hypothesis I, α diversity of Acidobacteria significantly changed with wetland degradation (Table 2; Figure 1), and the OTU, Shannon and Chao1 indices all showed the highest values in EW type and the lowest ones in MB. Since soil Acidobacteria in wetlands have been rarely studied, it is still unclear how the wetland vegetation affects the diversity of the soil Acidobacteria. However, from the results of this study, the EW type belongs to the transitional type with both wetland plants and forest plants, and its special habitat may be one of the reasons for the high diversity of its soil Acidobacteria. Another reason may be related to the overall bacterial diversity in soil, our previous study found that soil bacterial diversity was highest among EW types (Sui et al., 2021). Therefore, due to its high overall diversity, it may affect α diversity of Acidobacteria. However, it is worth noting that the Chao1 index in MB was the lowest but the Chao1 index of total bacteria was the highest (Sui et al., 2021). Naether et al. (2012) found that there were significant differences in the diversity of Acidobacteria between grasslands and forests, and even between different locations in the same ecological environment. Therefore, further in-depth research is needed in the future.

Our results indicated the Shannon index was significantly negatively correlated with soil TN and AN (Supplementary Table S2). However, we did not find that α diversity was significantly correlated with pH (Supplementary Table S2). Liu et al. (2014) reported that Acidobacteria in northeastern black soil farmland was significantly correlated with soil pH, and Fierer et al. (2007), Männistö et al. (2007), and Lauber et al. (2008) also pointed out that α diversity was significantly correlated with pH. However, the results of our research are not consistent with theirs. This is possible that the soil pH did not change significantly from wetland to forest (range from 5.5 to 5.8). In addition, we further found that the diversity of five Acidobacterial subgroups also highly correlated with soil pH (Figure 4). Our results reveal that soil pH acts as an effective habitat filter to restrict the Acidobacterial community in the Sanjiang wetland soils. Similar results were also reported by Jones et al. (2009), who indicated that Acidobacterial communities in soils were more phylogenetically clustered as pH departed from neutrality. Another reason is possible that Liu et al. (2014) study was based on a regional scale in northeast China, so the results may be in line with the distribution rule of microbial biogeography, that is, the distribution of soil microorganisms at a large scale is driven by pH. And because of a small scale of our study, the acidobacteriral diversity may be more significantly affected by soil nutrients. However, our previous study found that the soil Chao1 index under different vegetation types was the lowest in EW and the highest in MB (Sui et al., 2019), which was contrary to the result of Chao1 index of soil Acidobacteria. Different primers will lead to different results (Lee et al., 2008; Liu et al., 2014). Lee et al. (2008) found that the amplification efficiency of different primers for subgroup of Acidobacteria was different. The amplification efficiency of universal bacterial primers (515F/907R and 515F/806R) and Acidobacterial special primers (ACIDO/1492R, 31F/1492R, 341F/805R, and ACIDO/342R) for Acidobacteria is also different (Supplementary Table S3). Therefore, the diversity and distribution of the main bacterial phyla are not consistent, so further research is needed on the distribution of the diversity of different bacterial phyla in the wetlands of the Sanjiang Plain.

We hypothesized that changes in vegetation type resulted by wetland degradation would lead to changes in the composition of Acidobacteria. The proportion of Gp1, Gp2, Gp3 and Gp4 were higher in forest group than wetland group (Figure 3). Some studies showed that Acidobacterial composition was related to plant community types (Navarrete et al., 2013). Wang et al. (2010) found that Gp1, Gp2, Gp3, and Gp4 were dominant subgroups in forest soil. Naether et al. (2012) found that Gp4 and Gp6 were dominant subgroups in grassland soil. Yao et al. (2021) found that Gp4 and Gp6 were dominant subgroups in farmland soil. Therefore, the composition of Acidobacteria in the forest group in this study is consistent with the above studies. After the transition from wetland type to forest type, the wetland vegetation mainly composed of *Deyeuxia angustifolia* is transformed into broad-leaved forest and coniferous forest (e.g., *Betula platyphylla*, *Populus davidiana*, and *Larix gmelinii*), resulting the composition, litter, soil physicochemical properties were all changed, which may be the reason for the change in the abundance of the subgroup of Acidobacteria. Addition, Lee et al. (2008) proved that different primers lead to different amplification efficiencies for phylum Acidobacteria. Moreover, Supplementary Table S3 indicated that the use of different primers could affect the subgroup abundance of Acidobacteria. Even the different DNA extraction methods (e.g., CTAB and soil extraction kit) and sequencing methods (e.g., 454, Miseq and Hiseq) also influence the results of composition of Acidobacteria. Hence these may also be important reasons for the inconsistency between this study and other studies.

Through the correlation analysis between soil physicochemical properties and the community structure of soil Acidobacteria (Table 4), it can be found that the abundance of soil Acidobacterial community structure in the Sanjiang Plain is generally affected by soil available phosphorus and soil water content, but the forest group is mainly affected by soil pH, SOC, TN, AN, TP, AP, MC, while wetland types were mainly affected by TN, AN, and MC (Table 4). These are in accordance with our 2nd and 3rd hypothesis. Overall, our study is consistent with previous studies (Fierer et al., 2007; Jones et al., 2009), but in terms of forest type and wetland type, it seems to contradict to previous studies. Acidobacteria is an oligotrophic type of bacteria, mainly distributed in soils with low nutrient content (Fierer et al., 2007; Jones et al., 2009). However, a number of recent studies have shown that Acidobacteria can grow well under high nutrient conditions (Eichorst et al., 2007; de Castro et al., 2013). Navarrete et al. (2013) and Liu et al. (2014) proved the composition of Acidobacteria significantly positively related to soil organic carbon. Therefore, we speculate in the soil of the Sanjiang wetland type with high soil nutrient content, the relationship between the content of soil aciddobacteria and soil nutrients may have the following reasons. First, Acidobacteria is not correlated with soil organic carbon (Table 4), only Acidobacteria in forest types showed a significant positive correlation with soil organic carbon (Table 4). We found in a previous study that the total bacterial content in the Sanjiang wetland forest type were positively correlated with soil organic carbon (Sui et al., 2019), so this may be the reason of the positive correlation between Acidobacteria and soil organic carbon content. Second, it may be because there is a significant correlation between Acidobacterial subgroups in forest soil and soil nutrients. We found that Gp5 and Gp10 were significantly correlated with SOC (Figure 4). Naether et al. (2012) also found SOC had a positively relationship with soil Acidobacteria in grassland and forest soils.

At present, there were 28 subgroups in the Acidobacteria phylum (Barns et al., 2007). In this study, we found that there were 22 Acidobacterial subgroups in all soil samples, meaning that Acidobacteria is widely spread in the wetland soil of Sanjiang plain. Addition, Gp1, Gp2, Gp3, Gp4, Gp7, and Gp6 were the most abundance subgroups (Figures 2, 3; Supplementary Table S3). This result was consistent with other reports (Kielak et al., 2009; Wang et al., 2010; Liu et al., 2014; Yao et al., 2021). Liu et al. (2014) reported that the proportion of Gp1, Gp3, Gp4, Gp6 was higher in black soils in northeastern of China. Yao et al. (2021) also proved that the proportion of Gp4 and Gp6 was higher in farmland in northeastern of China. This indicated that Acidobacteria in black soil in northeastern China was dominated by Gp1, Gp3, Gp4, and Gp6, and our research is consistent with it. However, our study also found that the wetlands of the Sanjiang Plain also contain higher abundance of Gp2 and Gp7. Some studies showed that Gp7 was highly abundant in forests (Shen et al., 2013) and grasslands (Naether et al., 2012). However, we found that Gp7 was also high abundant in wetlands, so we speculate that Gp7 is can be widely distributed in different habitats. Navarrete et al. (2015) stated that the relative abundances of Gp7 were positively correlated with soil nutrient content, while the soil organic carbon of the Sanjiang Plain wetland was high, which may be the reason why the Sanjiang Plain wetland contains higher Gp7, but the reason of Gp7 could be adapted to different habitats is still unclear and further research is needed. Liu et al. (2014) found that Gp2 was very low in black soil in northeastern farmland, which seems to contradict this study. However, a large number of studies have shown that Gp2 is the main subgroup in forests (Jones et al., 2009; Wang et al., 2010; Wei et al., 2018), which is consistent with the results of this study because the abundance of Gp2 was highest in the forest group (Figure 3).

Through the correlation analysis between soil physiochemical properties and different subgroups of Acidobacteria (Figure 4), it can be found that the abundance of Acidobacteria in the Sanjiang Plain is closely related to soil organic carbon, total nitrogen, total phosphorus, available phosphorus, soil moisture content. Our hypothesis guess soil moisture is one of the key factors affecting Acidobacteria in wetlands. The soil moisture content of the wetlands in the Sanjiang Plain is high, and the soil moisture content fluctuates significantly with the change of rainfall, which is one of the main characteristics of wetland habitats. Moreover, Hartmann et al. (2017) found that soil moisture is the main environmental factor affecting Acidobacteria, which is consistent with this study. However, only Gp2, Gp16, Gp18, Gp19, and Gp25 showed significant correlation with soil pH (Figure 4). These findings suggest that the growth characteristics of different Acidobacterial subgroups are not identical. Some subgroups were more sensitive to soil pH, while others were sensitive to soil nutrient content (e.g., SOC, TN, TP and AP), probably because subgroups within Acidobacteria select different ecological niches (Hartmann et al., 2017). Acidobacteriaceae can be differentiated into copiotrophic or oligotrophic categories (Naether et al., 2012; Navarrete et al., 2015), so we speculate that this may be the reason that soil Acidobacteria was highly correlated with SOC, TN, TP, AP. It needs to be emphasized that since large-scale surveys on Acidobacterial abundances are rare, further studies are needed to reveal the relationships of abundances of different subgroups of Acidobacteria and soil physicochemical properties.

## Conclusion

To our knowledge, this is the first comprehensive study, using high-throughput sequencing, on Acidobacterial communities across wetland degradation stages. We found that Acidobacterial diversity changed significantly after vegetation conversion (from wetland to forests). Soil physicochemical properties were the key environmental factors regulating Acidobacterial communities, while the relative abundances of many Acidobacterial subgroups were dominantly controlled by soil water content, suggesting that the physiological characteristics of Acidobacteria are different at the subgroup level in wetland environment. We revealed that Gp1, Gp2, Gp3, Gp4, Gp6 and Gp7 were dominant in all vegetation types, while Gp5 was minor in all vegetation types in this study. Changes in soil water content and vegetation type following wetland degradation were confirmed as the primary factors in determining the diversity and distribution of Acidobacterial communities, which will further affect biogeochemical cycling and lead to changes in ecosystem functioning and service at the regional level.

## Data availability statement

The datasets presented in this study can be found in online repositories. The names of the repository/repositories and accession number(s) can be found in the article/Supplementary material.

## Author contributions

XS: designed and performed the experiment and prepared this manuscript. BF and M-HL: revised this manuscript and language editing. YL and RZ: helped to do the experiment and finish the bioinformatic analysis. HN and M-HL: designed this experiment. All coauthors contributed to manuscript editing. All authors have read and agreed to the published version of the manuscript.

## Funding

This work was funded by the Natural Sciences Foundation of Heilongjiang Province (LH2020C088); Heilongjiang Province Postdoctoral Research Start-up Fund Project (LBH-Q21167); the Outstanding Youth Foundation of Heilongjiang University (JCL202006); the China Scholarship Council Visiting Scholar Program (201908230401); National Key Research and Developmental Project of China (2016YFC0500405) and Key Project of Heilongjiang Province (GA19C006-6), Special Project Foundation of Heilongjiang Academy of Sciences (YZ202003); and the Basic Scientific Research of Provincial Higher Education Institutions in Heilongjiang Province of 2022. Open access funding was provided by the WSL—Swiss Federal Institute for Forest, Snow and Landscape Research.

## Conflict of interest

The authors declare that the research was conducted in the absence of any commercial or financial relationships that could be construed as a potential conflict of interest.

## Publisher’s note

All claims expressed in this article are solely those of the authors and do not necessarily represent those of their affiliated organizations, or those of the publisher, the editors and the reviewers. Any product that may be evaluated in this article, or claim that may be made by its manufacturer, is not guaranteed or endorsed by the publisher.

## Footnotes

^1^ http://www.usadellab.org/cms/?page=trimmomatic

^2^ http://drive5.com/aparse/
