# Erratum: Soil Acidobacterial community composition changes sensitively with wetland degradation in northeastern of China

**DOI:** 10.3389/fmicb.2023.1170284

**Published:** 2023-03-07

**Authors:** Frontiers Production Office

**Affiliations:** Frontiers Media SA, Lausanne, Switzerland

**Keywords:** Sanjiang plain, soil bacterial diversity, β diversity, high-throughput sequencing, forest, community structure

An omission to the funding section of the original article was made in error. The following sentence has been added: “Open access funding was provided by the WSL—Swiss Federal Institute for Forest, Snow and Landscape Research.”

The original article has been updated.

