# Supplementary material for: Soil Acidobacterial community composition changes sensitively with wetland degradation in northeastern of China
Source: Front Microbiol. 2022 Dec 23;13:1052161. doi: 10.3389/fmicb.2022.1052161 (PMC9816132; doi:10.3389/fmicb.2022.1052161)
Supplement: Supplementary file 1 [file Data_Sheet_1.doc]

Supplementary Material

# Supplementary Figures and Tables

Supplement Table 1. Vegetation types (composition, cover and height) along the successional gradient from flooded wetland to conifer forest

| Successional stage | Abbreviation | Plant composition | Cover  contribution (%) | Height  (cm) | Plant Shannon diversity index |
| --- | --- | --- | --- | --- | --- |
| Natural wetland | NW | *Deyeuxia angustifolia* | 90 | 105 | 0.44±0.02e |
|  |  | *Carex appendiculata* | 3 | 70 |  |
| Shrub-invaded wetland | IW | *Deyeuxia angustifolia* | 55 | 98 | 0.58±0.03c |
|  |  | *Spiraea salicifolia* | 30 | 112 |  |
| Shrub-dominated wetland | DW | *Spiraea salicifolia* | 50 | 120 | 0.47±0.02e |
|  |  | *Deyeuxia angustifolia* | 30 | 103 |  |
|  |  | *Anemone dichotoma* | 10 | 65 |  |
| Wetland edge | EW | *Deyeuxia angustifolia*  *Filipendula palmata*  *Galium aparine*  *Anemone dichotoma* | 85  5  2  2 | 98  43  57  68 | 0.53±0.02d |
| Young *Betula* forest | YB | *Betula platyphylla* | 90 | 900 | 0.88±0.04ab |
|  |  | *Populus davidiana* | 5 | 900 |  |
|  |  | *Ulmus macrocarpa* | 5 | 800 |  |
| Mature *Betula* forest | MB | *Betula platyphylla* | 85 | 1400 | 0.92±0.03a |
|  |  | *Populus sdavidiana* | 5 | 1400 |  |
|  |  | *Quercus mongolica* | 2 | 1550 |  |
|  |  | *Ulmu smacrocarpa* | 2 | 1100 |  |
| *Populus-Betula* mixed forest | PB | *Populus davidiana* | 60 | 800 | 0.85±0.04b |
|  |  | *Betula platyphylla* | 30 | 850 |  |
|  |  | *Quercus mongolica* | 2 | 1000 |  |
| Conifer forest | CF | *Larix gmelinii* | 100 | 900 | 0.54±0.03cd |

Plant Shannon diversity index (means ± 1 SD); different lowercases represent significant difference at *P* < 0.05 level, tested with Duncan multiple comparisons.

Supplement table 2 Correlation analyses between acidobacterial α diversity and soil physicochemical properties

|  | Chao1 | Shannon | OTU Richness |
| --- | --- | --- | --- |
| pH | -0.049 | 0.042 | -0.039 |
| SOC | **0.686**** | 0.385 | **0.679**** |
| TN | -0.213 | **-0.586**** | -0.143 |
| AN | -0.294 | **-0.555**** | -0.299 |
| TP | 0.358 | -0.14 | 0.261 |
| AP | **-0.461*** | -0.118 | **-0.454*** |
| MC | 0.265 | -0.349 | 0.246 |

** at the 0.01 level, * at the 0.05 level. Bold indicates significant correlation.

Supplement table 3 A comparison of relative abundance of the dominating acidobacterial subgroups in different ecosystems

| Site | Primer | Ecosystem | Relative abundance of the dominating acidobacterial  subgroup (%) | | | | | | | Reference |
| --- | --- | --- | --- | --- | --- | --- | --- | --- | --- | --- |
|  |  |  | Gp1 | Gp2 | Gp3 | Gp4 | Gp5 | Gp6 | Gp7 |  |
| China (Xishuangbanna) | 31F/1492R | Forest | 79 | 12 | 7 | 2 | - | - | - | Wang et al., 2010 |
| Germany (Schwabische Alb, Hainich, Schorfheide-  Chorin) | 31F/1492R | Forest | 26-85 | - | 7-11 | 6 | 12-13 | 1-14 | - | Naether et al.,  2012 |
|  |  | Grassland | - | - | 10 | 15 | 1 | 24 | 7 |  |
|  |  |  |  |  |  |  |  |  |  |  |
| China (Changbai Mountain) | 515F/907R | Forest | 52 | 29 | 8 | 2 | - | 2 | 2 | Shen et al., 2013 |
| China (Shennongjia Mountain) | 515F/806R | Evergreen broadleaved  forest | 7 | 6 | 6 | 12 | - | 49 | - | Zhang et al.,  2014 |
|  |  | Coniferous forest | 21 | 31 | 11 | 4 | - | 19 | - |  |
| Northeast China | ACIDO/342R | Farmland black soils | 23 |  | 17 | 24 | - | 30 |  | Liu et al (2016) |
| Northeast China | ACIDO/342R | Farmland black soils | - | - | - | 34-46 | - | 34-43 | - | Yao et al (2017) |
| China (southeast) | 341F/805R | Forest | 29-44 | 19-62 | 3-21 | 11-20 | 5-9 | 29-50 |  | Wei et al (2018) |
| Northeast China | ACIDO/342R | Wetland group | 24 | 2 | 8 | 3 | - | 4 | 6 | This study |
|  |  | Forest  group | 31 | 5 | 12 | 2 | - | 5 | 2 |  |


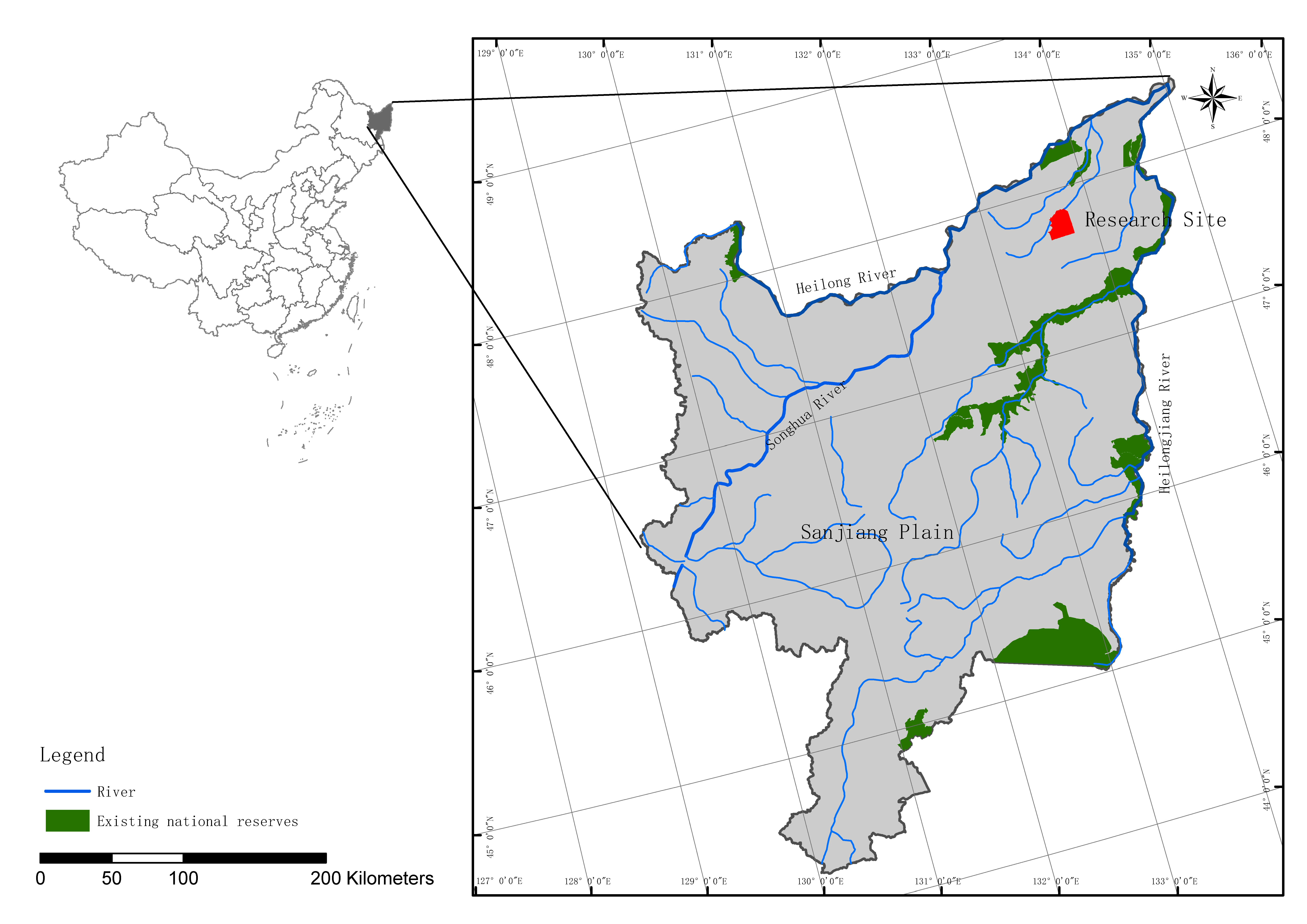


Supplement Figure 1. Study site location (red asterisk) in northeastern China.
